# Activation and proliferation profiles of *M.tuberculosis* specific dual functional CD4+T cells from smear negative pulmonary TB patients

**DOI:** 10.1371/journal.pone.0327243

**Published:** 2025-09-03

**Authors:** Ahmed Esmael, Adane Mihret, Tamrat Abebe, Daniel Mussa, Sebsib Neway, Joel Ernst, Jyothi Rengarajan, Liya Wassie, Rawleigh Howe

**Affiliations:** 1 Armauer Hansen Research Institute, Addis Ababa, Ethiopia; 2 Department of Microbiology, Immunology and Parasitology, College of Health Sciences, Addis Ababa University, Ethiopia; 3 Department of Medicine, Division of Infectious Diseases and Emory Vaccine Center, Emory University School of Medicine, Emory University, Atlanta, GeorgiaUnited States of America; 4 Division of Experimental Medicine, University of California San Francisco, San Francisco, California, United States of America; Centenary Institute, AUSTRALIA

## Abstract

**Background:**

Tuberculosis is a major public health challenge in the resource-limited endemic setting of sub-Saharan Africa. The diagnostic challenge becomes worse for smear-negative TB cases. Even if efforts for non-sputum-based TB diagnostic and prognostic biomarkers, there was limited data on blood-based immunological biomarkers among smear-negative PTB patients.Therefore, we assessed the phenotypic profile (HLA-DR, CD-38, Ki-67) of *M. tuberculosis* specific CD4 + T cells expressing dual IFN-γ and TNF-α cytokines from smear negative PTB patients in Addis Ababa, Ethiopia.

**Methodology:**

An institutional-based longitudinal cohort study was conducted in Addis Abeba, Ethiopia, on new smear-negative PTB who were adult and HIV-negative in comparison with multiple comparator groups. A total of 149 (confirmed patients with non-TB respiratory disease −33, smear-negative TB-29, smear-positive TB-34, apparently healthy – 53) study participants was enrolled. The expression level of activation (HLA-DR, CD-38) and proliferation (Ki-67) markers from dual IFN-γ and TNF-α cytokines expressing PPD specific CD4 + T cells were assessed after surface and intracellular cytokine staining. To confirm the presence of *M. tuberculosis*, MGIT/LJ culture, PCR, and smear microscopy were performed.

**Result:**

The overall level of HLA-DR and CD-38 expression in smear-negative and positive pulmonary TB patients were substantially higher than that of confirmed non-TB respiratory illness, apparently healthy QFT positive and negative study participants (p-value = 0.0127, p-value < 0.0001, p-value = 0.0043, p-value <0.0001, respectively) before commencing anti TB treatment. Also, among the smear-negative and positive pulmonary TB cohort, the expression of CD-38, HLA-DR, and HLA-DR + CD-38 + expression was reduced in the second month and six-month cohort compared with baseline data (p-value= < 0.0001, p-value = 0.00365, p –value = 0.0001, respectively).

**Conclusion:**

In this study, we found the diagnostic and prognostic potential of activation markers, particularly CD-38, in smear-negative PTB patients from dual *M. tuberculosis-specific* IFN-γ + TNF-α+ cytokine producing CD4 + T cells in both the presumed ex vivo and antigen-specific stimulation assays.

## Introduction

Globally, tuberculosis (TB) continues to be a significant public health issue [1]. One of the main factors is the effect of the Coronavirus disease 2019 (COVID-19), which resulted in a 13% rise in TB-related mortality and a 25% drop in TB detection. [1–3]. Another challenge to TB preventative measures and control programs is the difficulty in making an accurate diagnosis and predicting the effects of treatment promptly [4]. The existing tools cannot differentiate between latent and active TB, are time-consuming, and fail to meet clinical decision TB [5–10]. In this regard, smear-negative pulmonary TB (PTB) presents the greatest diagnostic difficulties. Also, a significant proportion of those cases (53.1% to 83%) were not etiologically confirmed [11–17]. Moreover, misdiagnosis and differential diagnosis with other respiratory diseases are the main challenges for clinicians [11–17].

More recently, the blood-based immune biomarkers; mostly the combination of phenotypic and functional markers of CD4 + T cells have received considerable attention as promising diagnostic, prognostic, and anti-TB treatment monitoring tools [18–22]. For example, the detection of *M. tuberculosis*-specific T cell cytokine response is the main reason behind the replacement of the Tuberculin skin test (TST) with Interferon-gamma release assays (IGRA) assay. Moreover, T cell activation and maturation marker (TAM) assay, which correlates activation profile with bacterial load, disease severity, degree of lung tissue destruction, and antigen clearance after anti-TB treatment proposed as a potential candidate to improve *M. tuberculosis* diagnosis and treatment monitoring [23–25]. Furthermore, many research efforts found the diagnostic and treatment monitoring potential of phenotype markers for smear-positive PTB patients. For instance, some studies associated polyfunctional (IFN-γ, TNF-α, IL-2) CD4 + T cells with latent TB infection [26], whereas others associated it with active TB. In addition, many studies have shown the diagnostic and prognostic potential of polyfunctional cells that express phenotypic markers, specifically HLA-DR and CD-38 with active TB [23,27–30]. Unfortunately, the heterogeneity in phenotypic and functional profiles throughout different stages of *M. tuberculosis* infection is a key hurdle to understanding distinct immune markers [26,28,31,32]. Moreover, phenotypic and functional immunological profiles in the Paucibacillary TB, smear-negative PTB cases are not explored in depth.

Unfortunately, there was limited data on paucibacillary TB, particularly among smear negative PTB patients. Therefore, in the present study, we sought to evaluate the phenotype profile (HLA-DR, CD-38, Ki-67) of *M.tuberculosis* specific CD4 + T cells producing dual IFN-γ and TNF-α cytokines from smear negative PTB patients before, during, and after completed anti-TB treatment in comparison patients with smear positive PTB, non TB other respiratory symptoms, apparently healthy QFT positive and negative study participants using polychromatic flow cytometry in Addis Ababa, Ethiopia. This could ultimately contribute to the existing knowledge on the efforts to evaluate the immune biomarkers for TB diagnostics, prognosis, and anti-TB treatment monitoring of *M.tuberculosis*.

## Methodology

### Study design, period and population

A facility-based longitudinal study was carried out in selected health facilities from August 2020 to February 10, 2021 in Addis Ababa, Ethiopia. Participants in the current study were Human Immune Deficiency Virus (HIV) negative, new adult smear positive, and negative pulmonary TB patients. In the present study, patients with confirmed non-TB other respiratory symptoms and participants who were apparently healthy but had either a positive or a negative QFT result were the comparator groups.. Participants with chronic debilitating conditions such as multi drug resistance TB (MDR-TB), extensive drug resistance TB (XDR-TB), and asthmatic patients were excluded for only the purpose of the study. Also, a total of 155 (confirmed patients with non-TB respiratory disease −33, smear-negative TB-31, smear-positive TB-34, apparently healthy – 57) study participants was enrolled in the present study. In this research context we defined study and comparator groups as follows

- **Smear negative PTB**: Cases who were new and negative by acid-fast bacilli (AFB) smear microscopy and positive culture in LJ/MGIT for *M. tuberculosis* or *M. tuberculosis* (RD9) Positive for PCR test.
- **Smear-positive PTB**: Patients who were newly diagnosed with TB upon AFB smear microscopy and showed growth in LJ/MGIT media or *M. tuberculosis* (RD9) were positive for PCR test.
- **Confirmed non-TB respiratory disease**: These were smear-negative TB suspects who responded to first-line broad-spectrum antibiotics treatment and should show no growth in LJ/MGIT for M. tuberculosis or M. tuberculosis (RD9) positive for PCR test. This group was followed for one month for their response to first-line broad-spectrum antibiotics. **Apparently healthy individuals: Thes**e **were healthy-looking individua**ls upon initial physical/clinical screening and who show no clinical signs/symptoms complex for TB and who lived in the same house with known TB patients, who later screened for latent TB infection using Quantiferon TB gold plus screening test and became positive (QFT+) or negative(QFT-).

### Laboratory activity and procedure

All participants were screened for HIV at their recruitment health facilities following the provider-initiative HIV counseling and testing (PIHCT) approach. Then, spot-spot sputum samples were collected aseptically from presumptive TB patients and controls. Furthermore, the study nurse or public health officer collected approximately 20 ml of venous blood. Finally, samples were transported to the AHRI laboratory at ambient temperature for further laboratory analyses. **PBMC isolation, stimulation, and staining:** In this study, all study participants who met the inclusion criteria initially screened for HIV counseling and testing. Then, 20 ml of whole blood was collected by trained clinical nurse or public health officer in the selected health facilities. For this immunological procedure, we used fresh PBMC, which isolated according to the standard operating procedure. In brief RPMI-1640 diluted whole blood layered on a leucosep tube (BD) containing 15 ml ficoll-paque TM plus (GE health care, endotoxin tested, density 1.077, lot- 10294423). Then, the fluid portion (2–3 ml) above PBMC was discarded and the PBMC was transferred into a new falcon (50 ml) tube. Following the subsequent wash with RPMI-1640, Cells re suspend with 1 ml complete media, counted, and dispensed into replicate 96-well culture plates, stimulated for 18 hours with Purified Protein Derivative (PPD)-10ug Serum Staten Institute) (10ug) and PHA-5ug from BEI resources. Brefeldin A (10 g/ml, Sigma, catalog No. 555029) was added at 30 minutes for PHA and 2 hours for *M. tuberculosis* peptides during stimulation. After 18 hours of stimulation, cells from a replicate 96-well plate were pooled, stained with a cocktail of surface anti-human monoclonal antibodies, and then incubated for 30 minutes. The cocktail included 2.5 ul each of the following antibodies: 2.5 ul CD38-BV421 (clone-HIT2 catalog No. 562444), 2.5 ul CD8-APC-CYTM7 (clone SK1 catalog No. 348813), 2.5 ul CD4-BV510 (clone SK3 catalog No.562970), and 2.5 ul HLA-DR-PE-CYTM7 (clone G46-6 catalog No. 560651) [33–35].

**Permeabilization, intracellular staining, and fixation**: After surface staining, cells were fix and permeabilize with cytofix/perm (BD, catalog No 512090kz) and stained with a cocktail of 5ul TNF-α-APC (clone-640.1111, catalog No. 340534), 5ul IFN-g-FITC (cloneB27, catalog No. 554700) and 5ul Ki-67-PerCP-CYTM5.5 (clone B56, catalog No. 561284) and incubated for 30 min. Then, washed two times with perm wash (catalog No 557885). Finally, cells were fixed with 300ul of 2% paraformaldehyde (PFA), washed, and resuspend with 500ul of FACS buffer until acquisition. Cells acquired using BD FACSCanto TM II, FACS DIVA TM software with a gate get set on mononuclear cells. [33–35].

**Quantiferon TB Gold assay:** This assay carried out according to the manual instructions provided with the kit assayed for IFN-gamma measurement using QFT R-plus ELISA (catalog number 622120, Germany, QIAGEN, Optical density 450 nm filter and 620nm reference filter, software softmaxRpro7.013) [33,34].

**Mycobacteriology laboratory**: In this study, we collected 5–10 ml of morning-morning sputum sample from TB patients and non TB patients with respiratory symptoms. N-acetyl L-cysteine- sodium hydro oxide method (NALC-NaOH) used to digest and processed sputum sample. Then, the sediment was inoculated into the *Mycobacterium* growth indicator tube/MGIT TM 960 (lot number, 0059457) Lowenstein–Jensen/LJ media. After heat, killing DNA was extracted using DNA extraction kit (69504 and 69506). We used different primers including RD9 PCR (RD-9 REV-RTPCR – 5`-CACTGCGGTCGCCATTG-3, TM-57–60OC, GC: 64.7%, 17 mer, RD9- FW- RTPCR- 5-TGCGGGCGGACAACTC-3, TM- 56–86OC, GC = 68.75%, 16mer, Eurofins genomics, H8223-24498/11) for *M.tuberculosis* identification. To ensure the quality of our work we run a negative control (media alone) and a positive control (PHA). Also, we carried compensation and calculated the compensation matrix for each experiment. Moreover, to minimize spillover across detectors, every safety measure has been considered. Moreover, we included a positive control (RH37V) and a negative control (RNase-free water) to ensure the quality of mycobacteriology laboratory work. *Data analysis*

The Armauer Hansen Research Institute data management center coded, entered, and cleaned socio-demographic and clinical data using SPSS version 25. FlowJo 9.9.6 version software was used to analyze all flow cytometric data in our study.Immunological responses (*M. tuberculosis*-specific, ex vivo response from unstimulated samples) comparison between groups (smear-positive TB, smear-negative TB, confirmed non-TB other respiratory group, apparently healthy QFT negative and positive) was analyzed using the non-parametric Mann-Whitney U test for baseline study. Additionally, the Wilcoxon matched paired signed rank test statistical analysis tool was used to compare the immunological responses at different time points (baseline, 2nd month, and 6th-month follow-up) within the group during the standard anti-TB treatment follow-up period. Also, all statistical analysis and graphical illustration made using GraphPad Prism version 6 software. The frequency of PPD-specific activation and proliferation markers from dual (IFN-γ + TNF-α) cytokine-producing CD4 T cells was calculated by subtracting the media from stimulated well and dual (IFN-γ + TNF-α) cytokine-producing CD4 T cells used as surrogate markers of antigen-specificity. The percentage of IFN-γ + TNF-α+ producing CD4 + T cells was always at least two-fold higher in the PPD stimulated samples than in the unstimulated (negative control) sample responseTo determine the fraction of activated or proliferating cells among PPD reactive T cells, we divided the above frequency of PPD- specific HLA-DR or CD-38 or Ki-67) positive cytokine produced by the aforementioned total PPD-specific cytokine-producing cells. Note that the term fraction here is mathematically equivalent to percentage/100.Statistical significance was defined as a p-value of less than 0.05.

### Ethical considerations

The Institutional Review Boards of Addis Ababa University, College of Health Sciences (AAU-CHS), and Armauer Hansen research institute/ All Africa Leprosy Rehabilitation and Training Center (AHRI/ALERT) Ethical Review Committee ethically examined and authorized this work. Before the study began, approval letters from the Addis Ababa Health Bureau and relevant institutions were obtained. All study participants provided written informed consent, and the information they provided was kept confidential. Pulmonary TB patients were given standard anti-TB drug treatments according to the national guideline.

## Result

### Socio-demographic characteristics of study participants

In the present study, a total of 155 (confirmed patients with non-TB respiratory disease −33, smear-negative TB-31, smear-positive TB-34, apparently healthy – 57) study participants was enrolled. Of apparently healthy participants, thirty became QFT positive, and twenty-three became QFT negative. Of those, six study participants including two smear negative culture negative PTB suspected patients who became RD9 negative and 4 apparently healthy study participants with indeterminate QFT result were excluded from main data analysis. The mean age of study participants was 34, with a standard deviation of 13. In the present study, 65 (42.5%) and 55 (34.9%) of the study participants consumed raw meat and milk, respectively. The majority of study participants, 83 (54.2%), were Amhara. 43 (28.1%) were in grades 5–8 in terms of education..

### Clinical symptoms, contact history, and Mycobacteriology result

The majority of TB patients and patients with confirmed non TB respiratory illness presented with a combination of symptoms, such as chronic cough, shortness of breath, production of sputum, fatigue/tiredness, unexplained weight loss, hemoptysis, and fever. Only 18 (11.8%) of the study’s participants had prior contact with individuals who were known to have TB. Of the patients with TB, only 16% had chest X-ray findings. The body mass index of the majority (BMI) of study participants was within the normal range. Of smear-positive TB patients, twelve patients had 3 + , nine patients had 2 + , and 13 patients had 1 + smear microscopy grade results. All of the smear-positive TB patients grew on LJ/MGIT and became RD9 positive. Additionally, only 12 smear-negative TB patients showed growth on LJ/MGIT, and 29 became RD9 positive while 2 became RD9 negative, and we excluded them from the final analysis.

### Flow cytometry gating strategy

**Fig 1:**
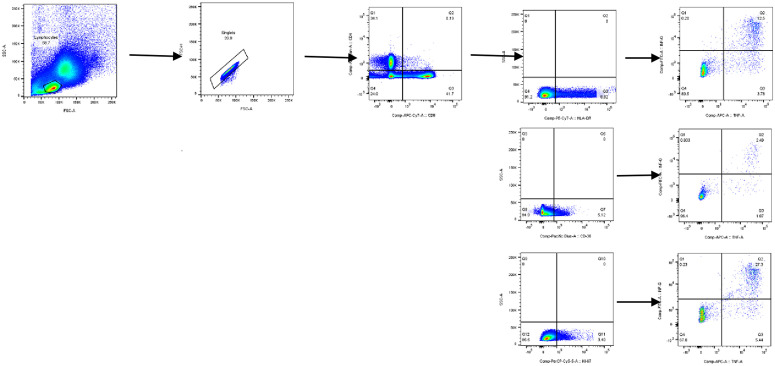
Gating strategy during the TB biomarkers study in selected health facilities in Addis Ababa, Ethiopia.

The forward scatter and side scatter properties were employed to gate lymphocytes. Doublets were excluded, and then CD4+ and CD8 + T cells were gated on. In this study, HLA-DR + /CD38 + /Ki-67 + T cells were first gated from PPD-specific CD4 + T cells, and then, from this population of cells, were (TNF-α + IFN-γ+ cells gated. The activation (HLA-DR, CD-38), and proliferation (KI-67) markers expression were analyzed on PPD specific dual (TNF-α + IFN-γ+) functional markers from CD4 + T cells population. In this study, the negative control was the background stain (i.e., stained but unstimulated). In the present study antigen specific stimulation profile for activation (HLA-DR, CD-38) and proliferation (KI-67) among pulmonary TB patients (smear positive and smear negative) and comparators (QFT positive, QFT negative, confirmed non TB other respiratory disease) were determined (Fig 1).

### Pattern of activation and proliferation markers expression from unstimulated dual IFN-γ + TNF-α+ producing CD4 + T cells from treatment naïve pulmonary TB patients

**Fig 2:**
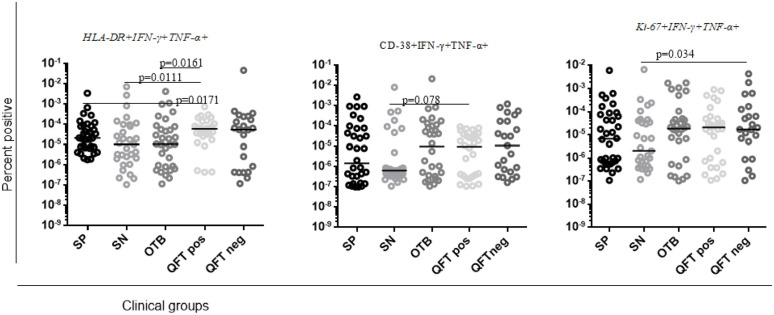
Percent positive of activation and proliferation profile from unstimulated dual IFN-γ + TNF-α+ producing CD4 + T cells. Non-parametric Mann–Whitney test was used to analyze data and P values < 0.05 were indicate. Legend labels “SN” to smear-negative pulmonary TB patients, “OTB” to patients with non-TB respiratory disease, “QFT Pos” to quantiferon positive (LTBI+), “QFT Neg: to quantiferon negative (LTBI−), “SP” refer to smear-positive pulmonary TB patients.

As shown in Fig 2, we noticed inconsistent levels of activation and proliferation markers (singly or in combination) from the unstimulated sample across PTB patients and comparator groups. The overall level of activation (HLA-DR, CD-38) and proliferation (Ki-67) markers in the smear-positive and negative pulmonary TB patients was much lower than compared with the healthy QFT positive and negative comparators groups (p-value = 0.0161, p = 0.0171,p = 0.0111, p = 0.034), respectively (Fig 2). In contrast, smear-positive PTB patients had higher levels of CD-38, HLA-DR, Ki-67, and HLA-DR + CD-38 + expression compared to smear-negative PTB patients, however, did not reach a substantial significance difference (Fig 2). Moreover, CD-38 and Ki-67 expressions from confirmed non-TB other respiratory illness patients were much higher compared to smear-negative PTB. Furthermore, the overall level of HLA-DR + Ki-67 + expression from smear-negative PTB patients was much lower compared with confirmed non-TB respiratory patients, apparently healthy QFT positive and negative study participants (Fig 2). On the other hand, the overall expression of CD-38 + Ki-67+ from confirmed non-TB other respiratory patients, apparently healthy QFT-positive and negative study participants were noticeably higher compared to smear-positive PTB patients and smear-negative PTB patients (Fig 2).

### Pattern of activation and proliferation profile of PPD specific dual IFN-γ + TNF-α+ producing CD4 + T cells from treatment naïve smear negative and positive pulmonary TB patients

**Fig 3:**
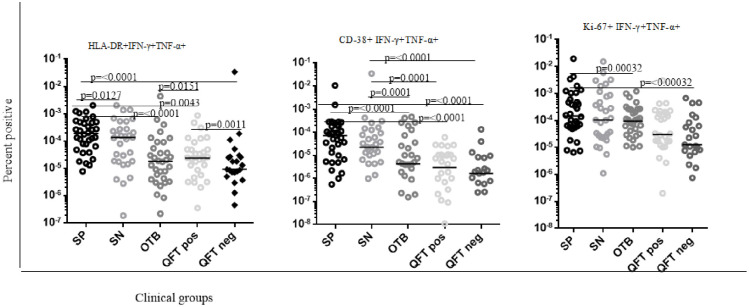
Pattern of activation and proliferation markers across different groups from PPD specific dual IFN-γ + TNF-α+ producing CD4 + T cells. For the statistical analysis, we used non-parametric Mann–Whitney test and P values < 0.05 were indicate. Legend labels “SN” to smear-negative pulmonary TB patients, “OTB” to patients with non-TB respiratory disease, “QFT Pos” to quantiferon positive (LTBI+), “QFT Neg: to quantiferon negative (LTBI−), “SP” refer to smear-positive pulmonary TB patients.

As depicted in Fig 3, the overall level of HLA-DR and CD-38 expression in smear-negative and positive pulmonary TB patients were much higher than that of confirmed non-TB respiratory illness, apparently healthy QFT positive and negative study participants (p-value = 0.0127, p-value < 0.0001, p-value = 0.0043, p-value <0.0001, respectively). Additionally, the overall level of HLA-DR, CD-38, and Ki-67 expression on PPD-specific dual IFN-γ + TNF-α + CD4 + Tcells from smear-positive pulmonary TB patients were higher compared with smear-negative pulmonary TB, confirmed non-TB respiratory disease, apparently healthy QFT positive and negative study participants (p-value = 0.0127, p-value = 0.0001, p-value = p-value < 0.0001, p-value < 0.0001, respectively). Additionally, the expression of HLA-DR, CD-38, and Ki-67 among apparently healthy QFT positive study participants was substantially higher compared with apparently QFT negative study participants (p-value = 0.0001), however inconsistent level was observed when compared with confirmed non-TB respiratory disease (Fig 3). Furthermore, although not statistically significant, the overall magnitude of activation markers (CD-38) from smear-positive pulmonary TB patients was higher than that of smear-negative pulmonary TB patients. In addition, the overall magnitude of CD-38 expression was considerably higher in both smear-positive and negative pulmonary TB patients compared with confirmed non-TB other respiratory patients, apparently healthy QFT-positive and negative study participants. Additionally, the overall expression of CD-38 among apparently healthy QFT-positive study participants was lower than that of confirmed non-TB other respiratory patients, unfortunately, it did not reach a statistically significance level. Additionally, the level of HLA-DR and CD-38 expression from confirmed non-TB respiratory patients were higher compared with apparently healthy QFT-negative study participants (Fig 3).

Furthermore, compared with smear-negative pulmonary TB, apparently healthy QFT negative, and QFT positive study participants had lower Ki-67 expression (p-value = 0.0001, p-value= < 0.0001, respectively). Similarly, the level of KI-67 expression from confirmed non-TB respiratory illness was higher than apparently healthy QFT positive and negative study participants, however comparable with smear-negative pulmonary TB patients, unfortunately, it did not reach a statistically significance level (Fig 3).

### Combination of activation and proliferation marker profile from PPD-specific dual IFN-γ + TNF-α+ producing CD4 + T cells from treatment naïve smear positive and negative pulmonary TB patients

The overall expression of HLA-DR + CD-38 and HLA-DR + Ki-67+ from double cytokine-producing PPD-specific CD4 + T cells among smear-negative and positive pulmonary TB patients was substantially higher compared with confirmed non-TB other respiratory illness, apparently healthy QFT positive and negative study participants (p-value = 0.00245, p-value = 0.0012, respectively) (data not shown).

Moreover, the overall level of CD-38 + Ki-67 + expressing PPD specific IFN-γ + TNF-α + CD4 + T cells from smear-positive pulmonary TB patients was much higher compared with smear-negative pulmonary TB patients (p value= < 0.0001), confirmed non-TB respiratory patients (p-value = < 0.0001), apparently healthy QFT positive and negative study participants (p value= < 0.0001, p value= < 0.0001, respectively). Furthermore, the overall combination of HLA-DR + CD-38+ and CD-38 + Ki-67+ from apparently healthy QFT-positive study participants was significantly higher compared with apparently healthy QFT-negative study participants. Moreover, the overall frequency of HLA-DR + Ki-67 + expressing PPD specific IFN-γ + TNF-α + CD4 + T cells from apparently healthy QFT-positive study participants was lower compared with confirmed non-TB other respiratory illness (data not shown).

### Change in activation and proliferation markers profile from the un stimulated samples on the smear positive and negative PTB patients during standard anti TB drug treatment follow up

From the unstimulated sample in the smear-negative PTB patients, we found the level of HLA-DR, CD-38, and Ki-67 expression from double positive cytokine-producing CD4 + Tcells during the six-month cohort were found higher compared with baseline data (p-value = 0.0345, p = 0.001, p = 0.042, respectively). On the other hand, the level of expression of CD-38 and Ki-67 in the second-month cohort was lower compared with both baseline and six-month cohorts from presumed in vivo stimulation. Also, the level of expression of HLA-DR + Ki-67+ from double positive cytokine-producing CD4 + T cells during the six-month cohort was higher compared with the baseline and second-month cohort (data not shown).

Furthermore, from the presumed in vivo stimulation, we observed considerably higher levels of activation and proliferation markers (as single or combination) from smear-positive PTB patients during the six-month cohort compared with both the baseline and second-month cohorts. On the other hand, we found inconsistent levels (comparable, increased, and decreased) of activation and proliferation markers between baseline and second-month anti-TB treatment cohort from the unstimulated samples (data not shown).

### Change in activation and proliferation markers profile from the PPD specific CD4 + T cells on the smear positive and negative PTB patients during standard anti TB drug treatment follow up

**Fig 4:**
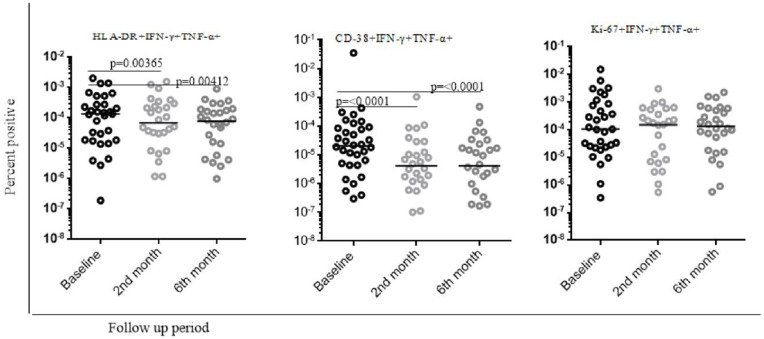
Overall level of activation and proliferation markers of PPD stimulated samples from smear negative pulmonary TB during anti TB treatment follow up in the selected health facilities in Addis Ababa, Ethiopia. In this study, non-parametric Wilcoxon matched-paired rank test was used to analyze data and P values < 0.05 were considered significant. Baseline represents time prior to initiation of standard anti TB treatment. 2nd and 6th month refers second and six month follow up after the initiation of standard anti TB treatment.

Following the standard anti-TB treatment among smear-negative PTB patients, we observed inconsistent levels of activation and proliferation markers expression from PPD-specific CD4 + T cells across different study cohorts, as explained in the following statement in detail. We found a slightly higher level of Ki-67 expression during the six-month cohort compared with baseline data from PPD-specific dual IFN-γ + TNF-α+ producing CD4 + T cells (Fig 4).. However, the level of Ki-67 expression during the second-month cohort was comparable with the six-month cohort. Also, among the smear-negative pulmonary TB cohort, the expression of CD-38, HLA-DR, and HLA-DR + CD-38 + expression was reduced in the second month and six-month cohort compared with baseline data (p-value= < 0.0001, p-value = 0.00365, p –value = 0.0001, respectively). The expression of HLA-DR + CD-38+ and HLA-DR + Ki-67+ in the second-month cohort was reduced compared with baseline data. However, it did not reach a statistically significant level. Also, comparable levels of expression of HLA-DR, CD-38, and Ki-67 were observed during the second and six-month cohorts from PPD-specific dual cytokine-producing PPD-specific CD4 + T cells (Fig 4).

**Fig 5:**
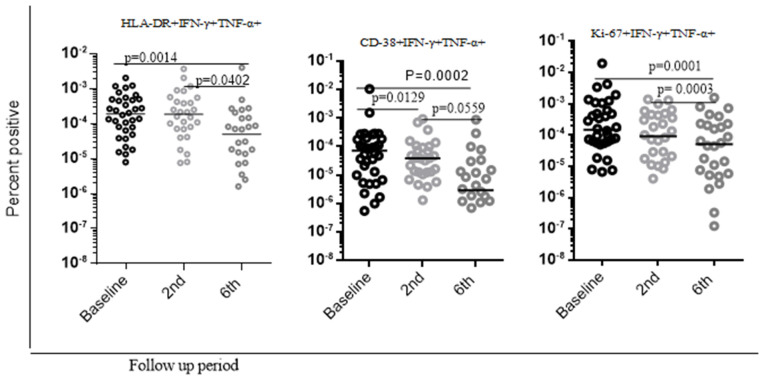
Overall level of activation and proliferation markers PPD stimulated samples from smear positive pulmonary TB during anti TB treatment follow up in the selected health facilities in Addis Ababa, Ethiopia. In this study, non-parametric Wilcoxon matched-paired rank test was used to analyze data. Baseline represents time prior to initiation of standard anti TB treatment. 2nd and 6th month refers second and six month follow up after the initiation of standard anti TB treatment.

Furthermore, among the smear-positive pulmonary TB patients, the level of activation (HLA-DR, CD-38, Ki-67) markers from PPD specific double positive cytokine secreting CD4 + T cells during the six-month cohort was significantly reduced compared with second and baseline data (p-value = 0.0014, p-value = 0.042, p-value = 0.0129, p-value = 0.0002, p-value = 0.0001, respectively). Also, in the case of the CD-38 markers, the level of activation in the second month significantly reduced compared with baseline data (p-value = 0.005). On the other hand, the level of HLA-DR and Ki-67 markers reduced in the second-month cohort compared with baseline data, however, It did not reach a statistically significant level (Fig 5).

Moreover, when we saw the level of combination of activation and proliferation markers in this cohort among smear-positive PTB patients, the level of combination of different activation and proliferation markers (HLA-DR + CD-38 + , HLA-DR + Ki-67 + , CD-38 + Ki-67+) in the six-month cohort significantly reduced compared with baseline data (p-value = 0.045, p-value = 0.0367, p-valu = 0.0478). The level of combination of those markers was also slightly reduced in the second-month cohort compared with baseline data even if not reach a statistically significant level (data not shown).

### Activation and proliferation markers profile between apparently healthy study participants and clinically cured pulmonary TB patients

In this study, we found a comparable level of activation and proliferation markers (in single and in combination) between apparently healthy QFT-positive study participants and six months of anti-TB treatment from smear-negative pulmonary TB patients (data did not show). Furthermore, a comparable level of activation and proliferation markers were found (HLA-DR, CD-38, and Ki-67) between QFT-positive study participants and six months of anti-TB treatment from smear-positive pulmonary TB patients. Moreover, the frequency of expression of activation and proliferation markers from the month anti-TB treatment cohort from both smear-positive and negative PTB was still higher compared with apparently healthy QFT-negative study participants (data not shownn).

## Discussion

Recently, much emphasis has been on the continuous *M. tuberculosis* metabolic spectrum over traditional dichotomous TB (i.e., latent and active TB) classification. This perspective gives a chance to define an additional clinical spectrum of TB such as incipient and sub-clinical TB [36]. Polychromatic flow cytometry may be used to characterize distinctive immunological profiles from different clinical spectra of *M. tuberculosis* infection, which might be helpful in the development of culture-free diagnostic and prognostic assays that could improve a better understanding of the complex *M. tuberculosis* immune response and improve TB diagnostics and prognostics. [37].

In this study, the smear-negative PTB patients showed substantially higher levels of HLA-DR, CD-38, and Ki-67 expression from M.tb specific dual IFN-γ + TNF-α+ producing CD4 + T cells than the apparently healthy QFT positive study participants. It would be ideal to anticipate a significantly higher level of activation and proliferation markers in smear-negative PTB than in apparently healthy QFT-positive. In our cases, we did not collect evidence regarding the hematological profile, X-ray, duration of disease/infection, and other co-morbidities, and those variables would modulate the level of activation and cell cycle proliferation. Also, when we compared these findings to our previous published studies, which evaluated the level of activation and proliferation markers from mono cytokine (IFN-γ/TNF-α+) producing PPD-specific CD4 + T cells, we found discrepancies in the level of activation and proliferation in the smear-negative PTB patients from dual cytokine ((IFN-γ + TNF-α+)) producing PPD specific CD4 + T cells. [33,34]. To address those differences, it is important to explore the biology of activation and proliferation markers (singly, in combination) about the mycobacterial load and *M. tuberculosis* stage-specific T cell profiles in the activation status. [38]. Moreover, different studies indicated the influence of opportunistic infections and co-infections on the modulation of the expression of immune activation. [39,40]. In this regard, our study found substantially higher levels of activation and proliferation markers in unstimulated samples in the comparator groups (confirmed non-TB other respiratory patients, apparently healthy QFT positive and negative study participants), which could be an indication for the presence of non-specific activation and proliferation in the comparator groups compared to the smear-negative PTB patients.Furthermore, when we used IFN-γ + TNF-α+ as an antigen-specific cytokine readout, our study demonstrated that CD-38 from PPD-specific CD4 + T cells had a high level of diagnostic accuracy in smear-negative PTB when compared to confirmed non-TB other respiratory patients, apparently healthy QFT positive and negative study participants. In line with this, many studies have shown that CD-38 has diagnostic potential in the T cell activation and maturation marker (TAM) assay, which could potentially be able to overcome the limitations of the currently available diagnostic tools [23–25]. On the other hand, Luo Ying and colleagues showed that HLA-DR had better diagnostic abilities than CD-38 from antigen-specific functional T cells. [29].

Following standard anti-TB therapy, we evaluated the level of dual (IFN-γ + TNF-α+) cytokine-producing CD4 + T cell activation and proliferation markers from *M. tuberculosis-specific*, smear-negative PTB patients. We found a substantial reduction in CD-38 in the second and six-month cohorts of smear-negative PTB patients during standard anti-TB treatment from *M. tuberculosis-specific* dual (IFN-γ + TNF-α+) cytokine-producing CD4 + T cells when compared to baseline data may indicate a potential prognostic role of this marker in these patients. This result was consistent with other studies, particularly that utilizing the T cell activation and maturation marker (TAM) assay. [23–25]. Also, from presumed ex vivo stimulation, we observed a substantially lower level of CD-38 from dual cytokine-producing CD4 + T cells in the second and six-month cohort compared with baseline data could support the prognostic potential not limited to antigen-specific stimulation, rather it would be applicable for the presumed ex vivo stimulation. Furthermore, the level of activation and proliferation markers expression from *M.tubeculosis* specific dual (IFN-γ + TNF-α+) cytokine-producing CD4 + T cells in the smear-positive PTB patients was significantly reduced in the six-month cohort compared with baseline and second-month cohort. This result was consistent with several studies that revealed the prognostic significance of such markers both during and after anti-TB therapy [23,41–44]. The difference in the level of activation between smear-negative and smear-positive PTB during standard anti-TB treatment could be related to bacillary load, cytokine/chemokine milieu, co-morbidity, co-infection, and predominance of the immune cell population. [23,29,33,41,44–46].

The key limitation of this study was the inability to quantify those activated and proliferated CD4 + T cells that were producing other cytokines due to the use of dual IFN-γ + TNF-α+ response as an antigen-specific readout. Additionally, we see a PPD-specific response in this case that might interact with other Mycobacterium TB complex types as well as other environmental Mycobacterium species. The other limitation in this study was the absence of hematological data, particularly monocytes or MLR (mixed lymphocyte reaction), which limits our ability to examine the impact of those variables on the expression of activation and proliferation T cell markers in smear-negative PTB patients in comparison with other comparator groups.To define the role of those activation markers in the diagnosis and prognosis of smear-negative PTB, further studies using *M. tuberculosis*-specific peptide pools could supplement our study
