# Correction: Activation and proliferation profiles of *M.tuberculosis* specific dual functional CD4+T cells from smear negative pulmonary TB patients

**DOI:** 10.1371/journal.pone.0338017

**Published:** 2025-12-02

**Authors:** Ahmed Esmael, Adane Mihret, Tamrat Abebe, Daniel Mussa, Sebsib Neway, Joel Ernst, Jyothi Rengarajan, Liya Wassie, Rawleigh Howe

There are errors in the author affiliations. The correct affiliations are as follows:

Ahmed Esmael^1^, Adane Mihret^1,2^, Tamrat Abebe^2^, Daniel Mussa^1^, Sebsib Neway^1^, Joel Ernst^3^, Jyothi Rengarajan^4^, Liya Wassie^1^, Rawleigh Howe^1^

1 Armauer Hansen Research Institute, Addis Ababa, Ethiopia, 2 Department of Microbiology, Immunology and Parasitology, College of Health Sciences, Addis Ababa University, Addis Ababa, Ethiopia, 3 Division of Experimental Medicine, University of California San Francisco, San Francisco, California, USA, 4 Department of Medicine, Division of Infectious Diseases and Emory Vaccine Center, Emory University School of Medicine, Emory University, Atlanta, Georgia, USA.
